# Automatic generation of smart earthquake-resistant building system: Hybrid system of base-isolation and building-connection

**DOI:** 10.1016/j.heliyon.2016.e00069

**Published:** 2016-02-03

**Authors:** M. Kasagi, K. Fujita, M. Tsuji, I. Takewaki

**Affiliations:** Dept. of Architecture and Architectural Eng., Kyoto University, Nishikyo, Kyoto 615-8540, Japan

**Keywords:** Engineering, Building, Mechanics, Structural engineering, Civil engineering

## Abstract

A base-isolated building may sometimes exhibit an undesirable large response to a long-duration, long-period earthquake ground motion and a connected building system without base-isolation may show a large response to a near-fault (rather high-frequency) earthquake ground motion. To overcome both deficiencies, a new hybrid control system of base-isolation and building-connection is proposed and investigated. In this new hybrid building system, a base-isolated building is connected to a stiffer free wall with oil dampers. It has been demonstrated in a preliminary research that the proposed hybrid system is effective both for near-fault (rather high-frequency) and long-duration, long-period earthquake ground motions and has sufficient redundancy and robustness for a broad range of earthquake ground motions.An automatic generation algorithm of this kind of smart structures of base-isolation and building-connection hybrid systems is presented in this paper. It is shown that, while the proposed algorithm does not work well in a building without the connecting-damper system, it works well in the proposed smart hybrid system with the connecting damper system.

## 1 Introduction

In the seismic resistant design of building structures, the concept of resilience is becoming more and more important and it is highly desired to design building structures safely for a broader class of possible earthquake ground motions (Takewaki, 2006, Takewaki, 2013; Takewaki et al., 2012). This is based on the common understanding that earthquake ground motions are highly uncertain both in its occurrence and property. It appears therefore difficult to predict the forthcoming events precisely in time, space and character (Takewaki, 2006, Takewaki, 2013; Takewaki et al., 2011, Takewaki et al., 2012, Takewaki et al., 2013). It is also recognized that the properties of building structural elements (especially the properties of base-isolation systems and passive control systems, etc.) are not deterministic (Ben-Haim, 2001) and their variation brings various difficulties in the seismic resistant design of building structures in terms of robustness and redundancy (AIJ, 2011). In fact, it is code-specified in Japan to take into account the variability of mechanical properties of isolators and dampers in the design of base-isolated buildings and passively controlled buildings. In this design process, the worst combination of mechanical properties of isolators and dampers is investigated (Ben-Haim 2001; Elishakoff and Ohsaki, 2010; Takewaki et al., 2012) and all the design conditions are checked for this worst case.

While base-isolated buildings are understood to be effective for high-frequency (impulsive) ground motions and base-isolation systems were used for rather rigid super structures in an early stage (Jangid and Datta, 1994; Hall et al., 1995; Heaton et al., 1995; Jangid, 1995; Jangid and Banerji, 1998; Kelly, 1999; Naeim and Kelly, 1999; Jangid and Kelly, 2001; Morales, 2003; Takewaki, 2005; Li and Wu, 2006; Takewaki, 2008; Hino et al., 2008; Takewaki and Fujita, 2009), they are not necessarily resistant for long-period ground motions with the characteristic period of 5–8s (Ariga et al., 2006; Irikura et al., 2004; Kamae et al., 2004). Actually it is reported that a resonance of the base-isolated buildings with the long-period ground motions was observed during the 2011 Tohoku (Japan) earthquake. Nevertheless, the base-isolation system is used recently even for a rather tall building especially in Japan and the long-period ground motions with the characteristic period of 5-8s are of great interest in the structural design of base-isolated buildings and super high-rise buildings. On the other hand, it is also well understood that, while building structures including passive energy dissipating systems are effective for long-duration, long-period ground motions (Takewaki, 2007, Takewaki, 2015; Patel and Jangid, 2011; Takewaki et al., 2011, Takewaki et al., 2012), they are not necessarily effective for near-fault (rather high-frequency) ground motions (Xu et al., 2007; Takewaki and Tsujimoto, 2011). This is because the passive damper systems cannot respond effectively to impulsive loadings. Smart resolution of these two issues may be one of the most controversial issues in the field of seismic resistant and control design (Koo et al., 2009; Petti et al., 2010; Karabork, 2011).

In this paper, a new hybrid passive control system is investigated in which a base-isolated building is connected to another non-base-isolated building (free wall) with oil dampers. A similar type of buildings without base-isolation is being designed and constructed by Obayashi Corporation in Japan as an apartment house with a car parking tower (Nishimura et al., 2008) and buildings incorporating such hybrid system are under construction in Tokyo by Obayashi Corporation and Shimizu Corporation. It may also be interesting to note that, if the free wall becomes stiffer, the connecting damper system yields a building with dampers attached directly to the ground (Trombetti and Silvestri, 2004). A preliminary investigation was conducted by Murase et al. (2013). However it seems that more detailed and deeper investigations are required. An automatic generation algorithm of this kind of smart structures of base-isolation and building-connection hybrid systems is presented in this paper.

## 2 Model

### Base-isolated building interconnected to outer frame

**Fig. 1:**
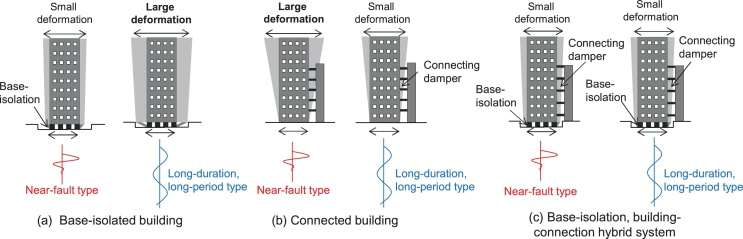
Earthquake response properties under near-fault and long-duration, long-period ground motions: (a) Base-isolated building, (b) Connected building, (c) Base-isolation, building-connection hybrid system.

**Fig. 2:**
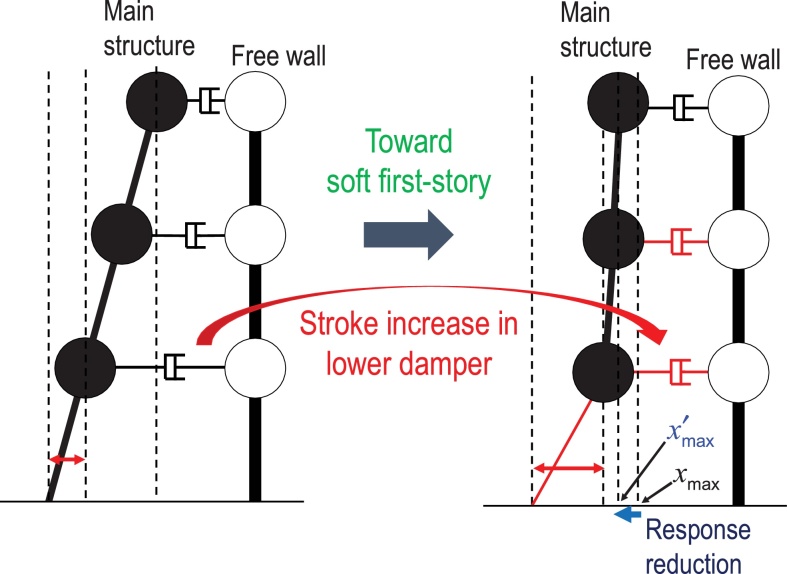
Advantageous feature of base-isolation for the energy consumption at the connecting dampers (*x*_max_: top displacement of initial model, *x*′_max_: top displacement of hybrid system).

A base-isolated building and a building with connecting dampers are two representatives of passive controlled buildings (see Figs. 1(a), (b)). A new hybrid passive control system consists of a multi-story base-isolated main building (apartment house), a free wall (car parking tower) and a group of interconnecting oil dampers as shown in Fig. 1(c). Oil dampers are usually installed as connecting dampers because of its sufficient stroke and damping performance. The general earthquake response properties of these buildings under near-fault and long-duration, long-period ground motions are explained in Fig. 1. While the base-isolated building is vulnerable to the long-period ground motion and the connected building is vulnerable to the near-fault ground motion, the building with the proposed hybrid system is effective for both types of ground motions. The hybrid passive control system can resist for the near-fault ground motion via the base-isolation mechanism and respond effectively to the long-duration, long-period ground motion via the building connection mechanism. Furthermore, the base-isolation mechanism is quite advantageous for the energy consumption at the connecting dampers in all stories as shown in Fig. 2.

## 3 Theory

### Automatic generation algorithm of hybrid control system

In this section, an automatic generation algorithm of the proposed base-isolation and building-connection hybrid system is presented. The main purpose of passive control systems is to reduce the acceleration and deformation of the main buildings. Let $y_{\max}^{\text{DIS}}$ and $y_{\max}^{\text{ACC}}$ denote the maximum top-mass displacement relative to the ground and acceleration of the main building and let $y_{\max}^{\text{DIS}(ini)}$ and $y_{\max}^{\text{ACC}(ini)}$ denote the maximum top-mass displacement and acceleration of the main building for the initial model. Although it is recognized in general that, as the building stiffness becomes smaller, the acceleration becomes smaller and the displacement becomes larger (see Section 4.3), this does not apply for the base-isolated building with the connecting-damper system. As seen in the numerical examples in the following section, the base-isolated building can make the top acceleration smaller with the top displacement almost constant. It may be useful to introduce the following objective function in terms of deformation reduction and acceleration reduction indices.

(1) $$f(y_{\max}^{DIS},y_{\max}^{ACC})=a\frac{y_{\max}^{DIS}}{y_{\max}^{DIS(ini)}}+b\frac{y_{\max}^{ACC}}{y_{\max}^{ACC\;(ini)}}$$

where *a* and *b* are the weighting coefficients.

In order to obtain a better design with a lower objective function, a sensitivity-based method is introduced. The stiffness of the free wall is fixed and only the stiffness of the main structure is treated as the design variable. The isolation-story stiffness can also be treated as a design variable by regarding this story stiffness as the isolation-story stiffness (insert of the isolation system at any floor is possible). If the gradient-based algorithm is not used, the structural designer cannot find directly the most appropriate location to decrease the stiffness at the first stage. Furthermore each set of stiffnesses of the main structure at each step obtained by the gradient-based algorithm provides the structural designers with useful information on structural design (the efficient location of stiffness). One of the popular sensitivity-free methods is GA (genetic algorithm). When GA is used, a complicated setting of GA parameters is necessary and this procedure may be cumbersome for most of structural designers.

The algorithm of the proposed method is very simple and can be summarized as follows.

1. Model the connecting building system into *N*-story shear buildings with connecting dampers
2. Consider *N* candidates with a slightly reduced story stiffness in only one story
3. Compute the responses of the above *N* candidates under a design ground motion and evaluate the objective function in terms of top acceleration and top displacement
4. Find the design with the lowest objective function among the above *N* candidates (the stiffness reduction is alternatively applied to each floor)
5. If the smallest story stiffness is violating a determined lower constraint, stop the procedure. Otherwise return to [Step 2].

**Fig. 3:**
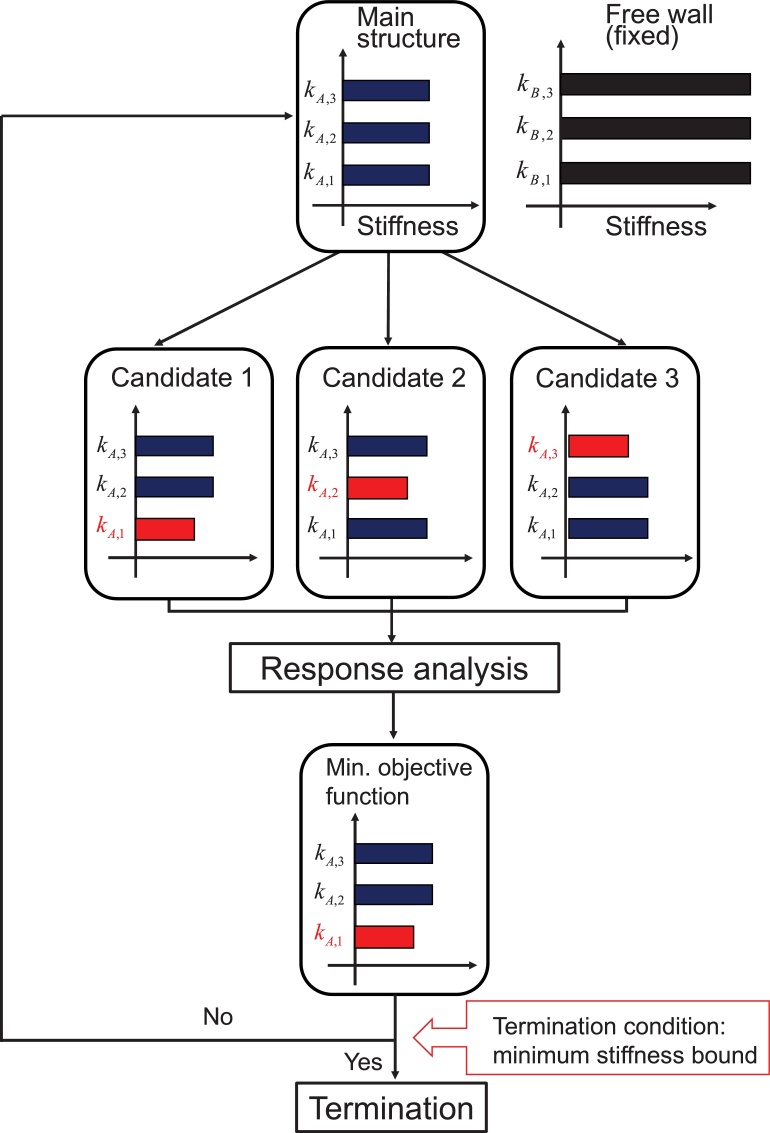
Schematic diagram of sensitivity-based automatic generation algorithm of smart hybrid system.

Fig. 3 shows the schematic diagram of the proposed sensitivity-based automatic generation algorithm of the proposed smart hybrid systems.

## 4 Example

In order to demonstrate the validity of the proposed method, some numerical examples are shown in this section. As stated before, the stiffness of the free wall is fixed and only the stiffness of the main structure is treated as the design variable.

### 4.1 Earthquake ground motions

**Fig. 4:**
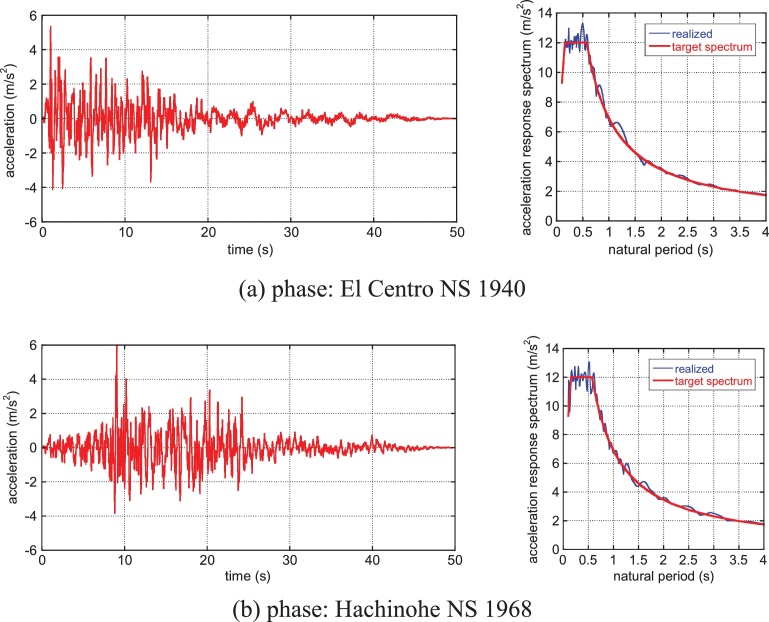
Design earthquake ground motions compatible with the design response spectrum in Japan: (a) Phase of El Centro NS 1940, (b) Phase of Hachinohe NS 1968.

The general properties of this hybrid system under near-fault (rather high-frequency) and long-duration, long-period ground motions have been disclosed in the previous work (Murase et al., 2013). In this paper, general design ground motions compatible with a specific code-specified design response spectrum in Japan is used. These ground motions are used in Japan for the design of high-rise buildings and base-isolated buildings. Two representative phase properties are employed to represent the two types of ground motions, i.e. El Centro NS 1940 for the near-field (impulsive) ground motion and Hachinohe NS 1968 for the far-field (long-duration) ground motion. Fig. 4 shows the acceleration time history and the acceleration response spectrum with the code-specified design acceleration response spectrum in Japan.

### 4.2 Hybrid building system

Consider a 30-story building with the hybrid system. The parameters of the original main building and the original free wall are shown in Table 1. For simple investigation, the building is condensed into a three-mass model. The parameters of such simplified three-mass model (mass and story stiffness) are presented in Table 2. It should be noted that, although the original model has been reduced to a three-mass model and the terminology of ‘story’ may not be appropriate, the expression ‘story’ has been used here. This is because it is intended to demonstrate, if the reduction of stiffness (insertion of base-isolation story) is effective, which floor level is most effective. The insertion of base-isolation story (insertion into one story of the original MDOF model) induces the reduction of the stiffness of the reduced element including the base-isolation story. Since it is useful to indicate the level of the element in the reduced model, this terminology ‘story’ is used in the sense of element. The structural damping ratios of the main structure and the free wall are 0.03 as stated in Table 1 for the original models. The connecting dampers are located uniformly at every mass level. The effect of higher modes will be discussed in Section 4.5 using the transfer functions.

The initial model of the main building is designed so as to have the fundamental natural period of 2.4(s) and a straight-line fundamental mode. On the other hand, the free wall is designed so as to have the fundamental natural period of 1.0(s) and a straight-line fundamental mode. The parameters in Eq. (1) are specified as *a* = 1, *b* = 1, i.e. the objective function is a simple sum of top displacement ratio and top acceleration ratio.

**Fig. 5:**
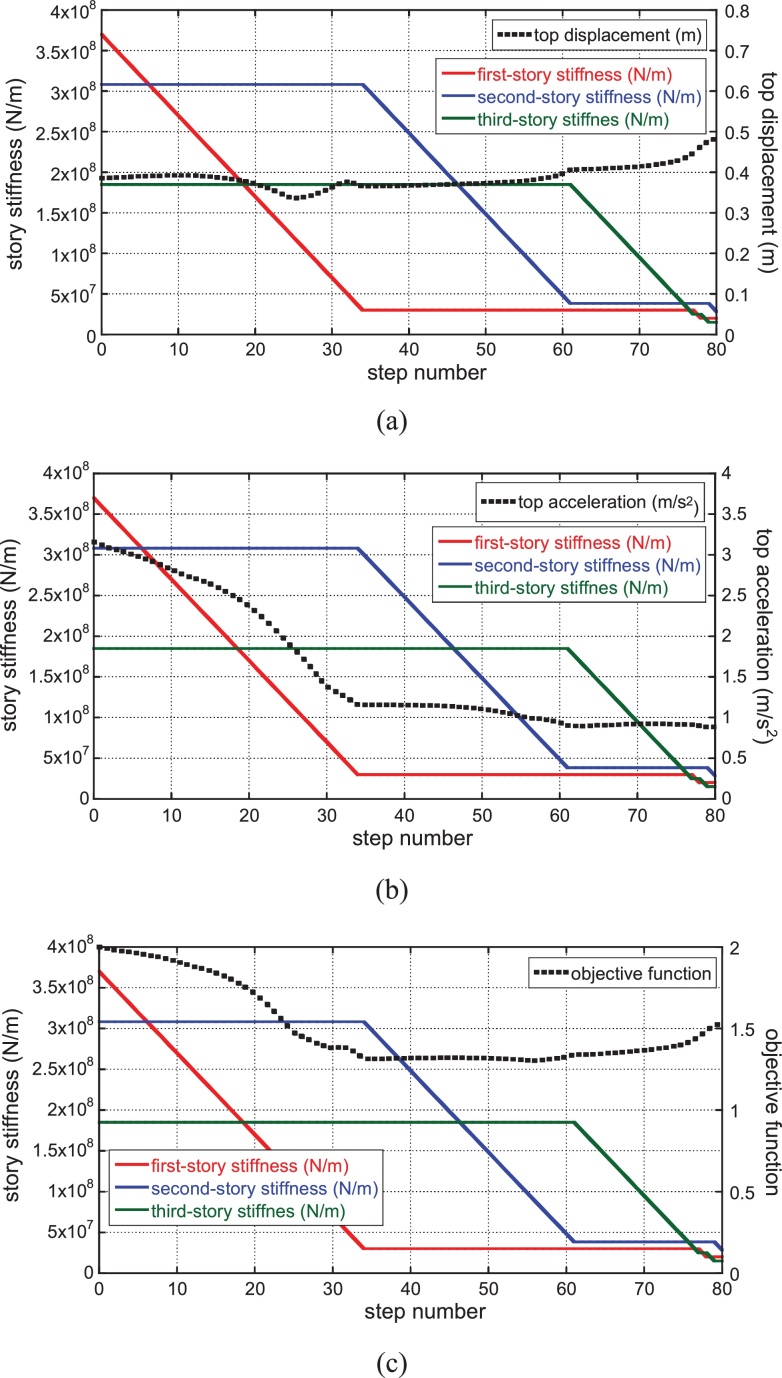
Transition of story stiffness and variation of top displacement, top acceleration and objective function for gradual decrease of main structure stiffness: (a) top displacement, (b) top acceleration, (c) objective function (Phase: El Centro NS 1940) (‘story’ is used here to designate the element of stiffness in reduced three-mass model).

Fig. 5 shows the transition of story stiffness and the variation of top displacement, top acceleration and objective function (linear combination of top displacement and top acceleration) for gradual decrease of the main structure stiffness. The sum of damping coefficients of connecting dampers has been given as 3.0 × 10^7^ (N/(m/s)) after some investigations. The damping ratio of the connecting dampers corresponding to the initial design is 0.16. This value has been derived from the actual design of an apartment building in Tokyo by Obayashi Corporation. The phase of the ground motion is that of El Centro NS 1940. Since the parameters in Eq. (1) are specified as *a* = 1, *b* = 1, the objective function for the initial model is 2.0. The variations of top displacement and top acceleration are also plotted at each step in order to monitor the response. It can be observed that, while the proposed algorithm does not work well in the building without the connecting-damper system (see Section 4.3), it works well in the proposed smart hybrid system with the connecting damper system.

**Fig. 6:**
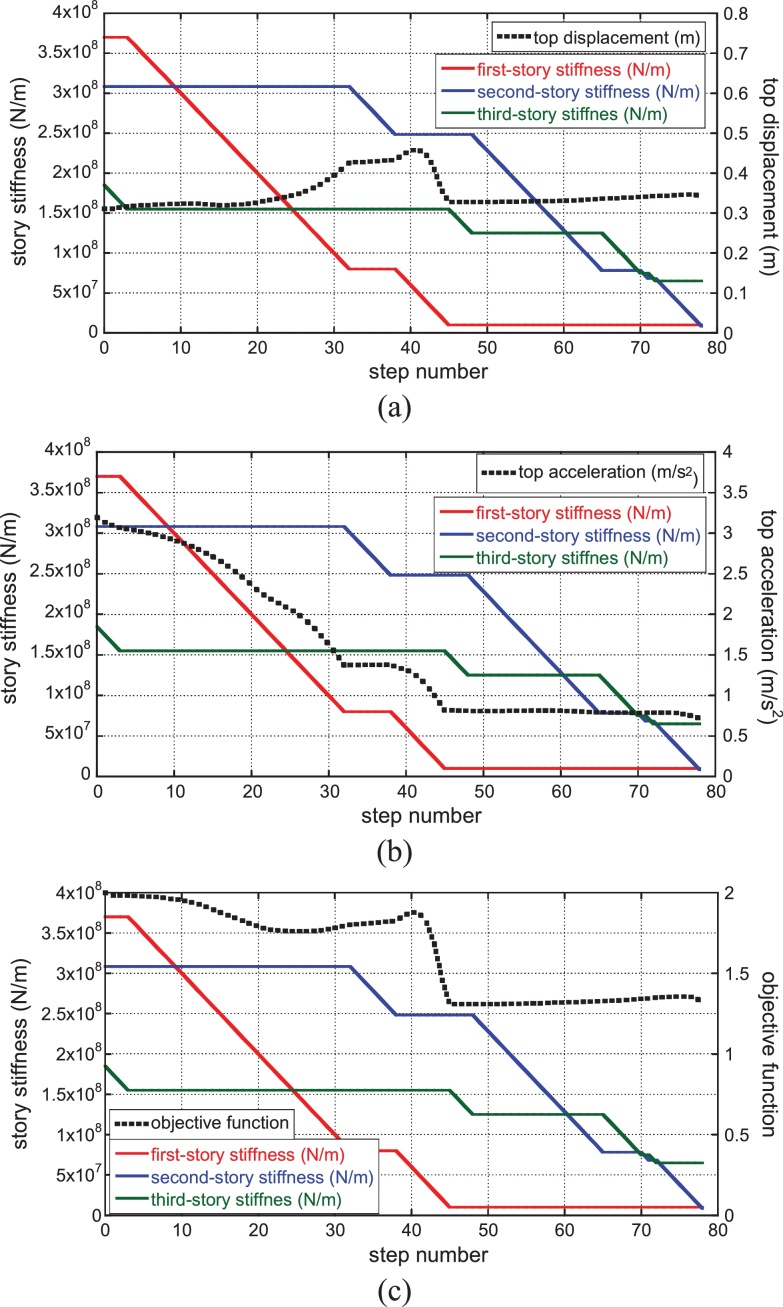
Transition of story stiffness and variation of top displacement, top acceleration and objective function for gradual decrease of main structure stiffness: (a) top displacement, (b) top acceleration, (c) objective function (Phase: Hachinohe NS 1968) (‘story’ is used here to designate the element of stiffness in reduced three-mass model).

On the other hand, Fig. 6 shows the corresponding figures for the ground motion with the phase of Hachinohe NS 1968. It can be understood that a similar transition of story stiffness is seen regardless of the variation of ground motions.

**Fig. 7:**
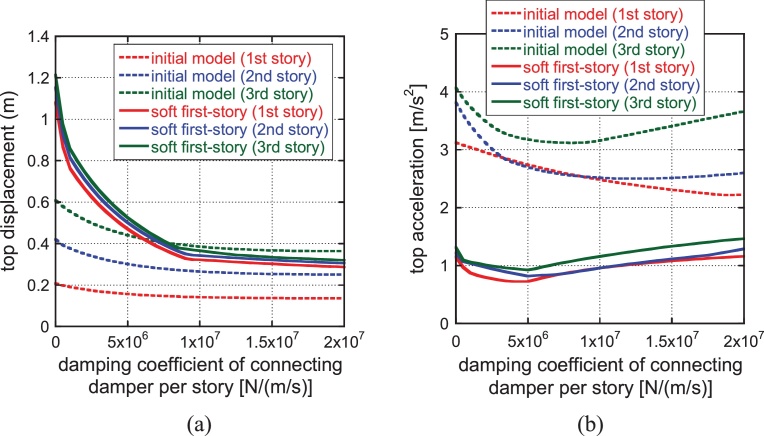
Variation of response with respect to damping coefficient of connecting damper for the initial model and the automatically generated soft first-story model (Phase: El Centro NS 1940): (a) Top displacement, (b) Top acceleration (‘story’ is used here to designate the element of stiffness in reduced three-mass model).

Fig. 7 shows the variation of top displacement and top acceleration with respect to the damping coefficient of the connecting damper for the initial model and the automatically generated soft first-story model under the ground motion with the phase of El Centro NS 1940. The damping coefficient 1.0 × 10^7^ (N/(m/s)) in each story corresponds to the sum 3.0 × 10^7^ (N/(m/s))of damping coefficients of connecting dampers employed in Section 4.2. As stated before, the connecting dampers are located uniformly at every mass level. It can be observed that the damping coefficient 1.0 × 10^7^ (N/(m/s)) exhibits a desirable response reduction both in the top displacement and top acceleration. As for the optimal damper location, in the proposed hybrid system the damper performance is not affected much by the damper distribution because the base-isolation induces large horizontal displacements at all the mass levels.

**Fig. 8:**
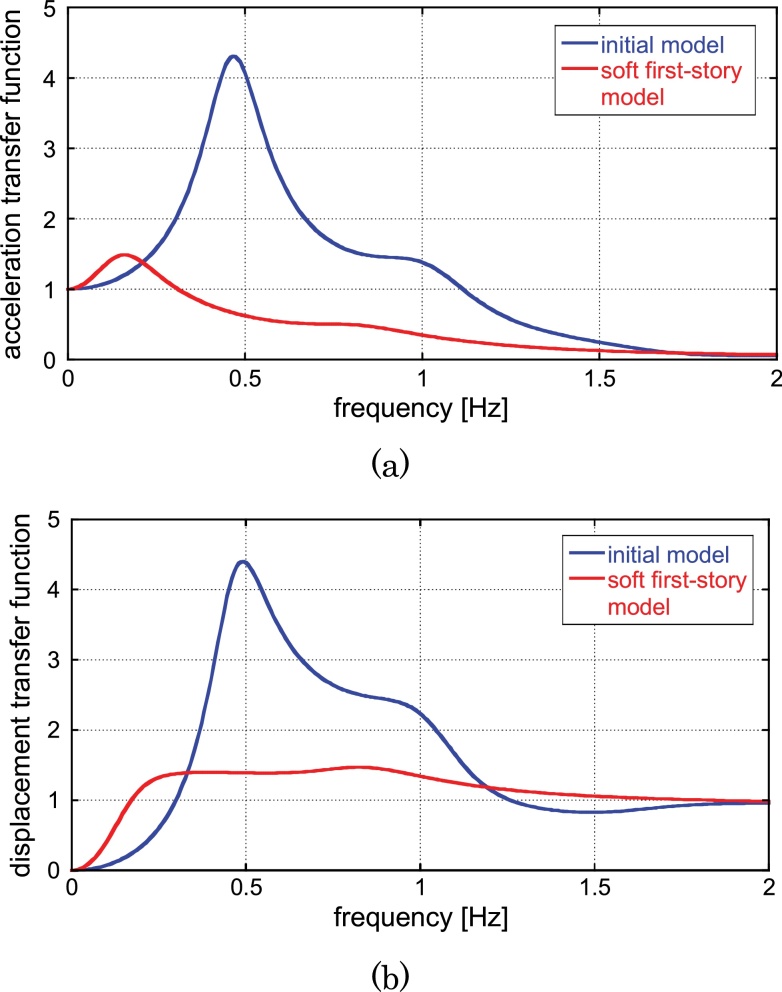
Acceleration and displacement transfer functions for initial model of uniform story stiffness and automatically generated soft first-story model: (a) Acceleration, (b) Displacement.

Fig. 8 illustrates the acceleration and displacement transfer functions for the initial model of uniform story stiffness and the automatically generated soft first-story model. It can be seen that the soft first-story model exhibits an excellent performance in the acceleration and displacement transfer functions.

**Fig. 9:**
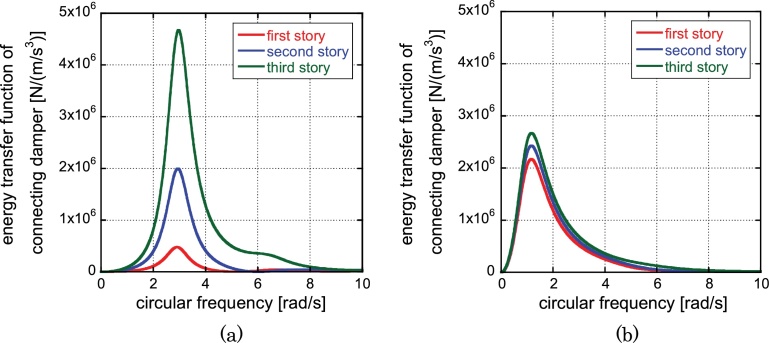
Energy transfer functions of connecting dampers in the 1st, 2nd and 3rd stories for the initial model and the automatically generated soft first-story model: (a) Initial model, (b) Soft first-story model (‘story’ is used here to designate the element of stiffness in reduced three-mass model).

The energy transfer function as a general transfer function for energy input is an effective index for demonstrating the energy absorption capacity of structural elements (Takewaki 2004, 2007, 2015). The energy transfer function can be obtained by applying the Fourier transformation and the inverse Fourier transformation to the expression of the total earthquake input energy in time domain. The integration in frequency domain of the energy transfer function multiplied by the squared Fourier amplitude of the input ground acceleration provides the total input energy. The area of the energy transfer function in frequency domain is relating directly to the input energy under the ground motion with the constant Fourier amplitude. Fig. 9 shows the energy transfer functions of the connecting dampers in the 1st, 2nd and 3rd stories for the initial model (Fig. 9(a)) and the soft first-story model (Fig. 9(b)). It can be observed that, while a large variability exists in the initial model, a fairly common distribution is realized in the soft first-story model. This indicates that the soft first-story model enables the uniform energy consumption at the connecting damper in every story.

### 4.3 Application of sensitivity-based algorithm to non-connecting building

**Fig. 10:**
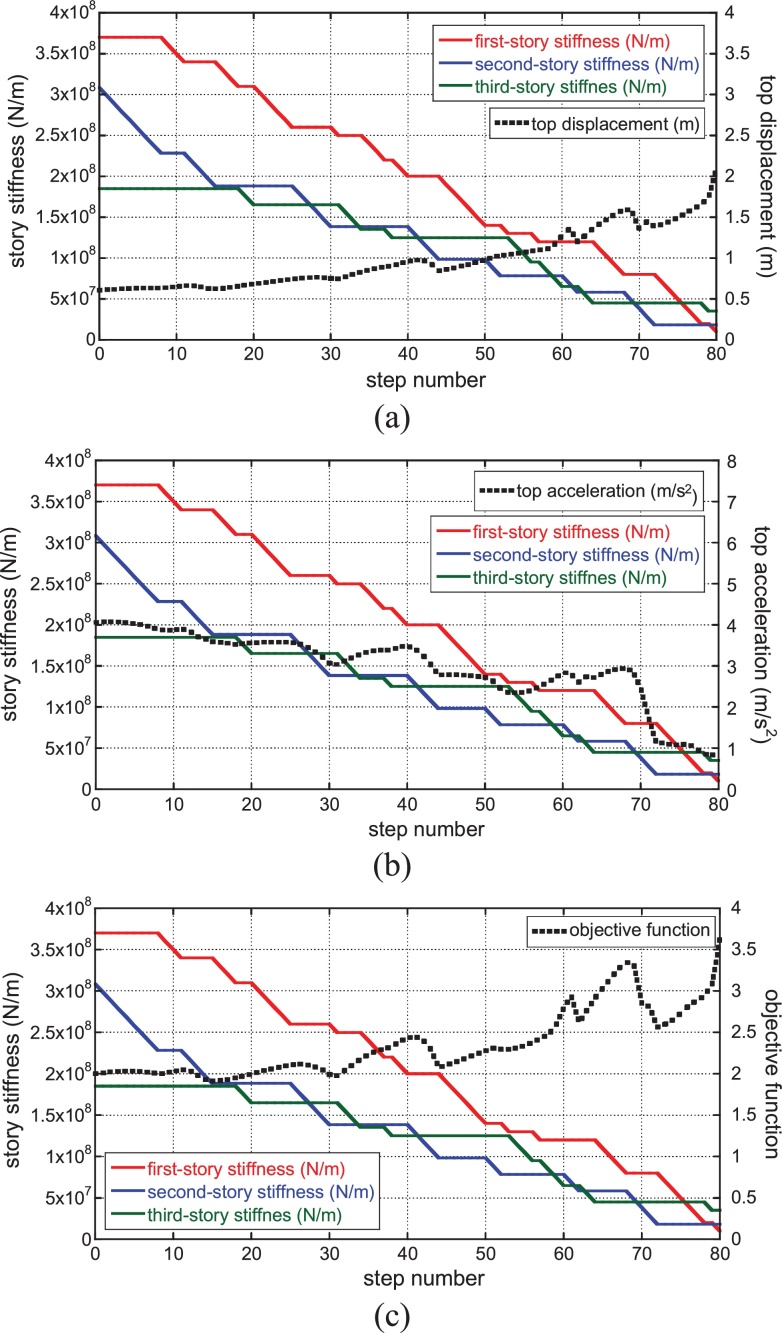
Transition of story stiffness and variation of top displacement, top acceleration and objective function for non-connecting building (linear combination of top displacement and top acceleration) (Phase: El Centro NS 1940) (‘story’ is used here to designate the element of stiffness in reduced three-mass model).

In order to investigate the applicability of the present sensitivity-based algorithm to non-connecting buildings, consider the same main structure as treated in Section 4.2. Fig. 10 shows the transition of story stiffness and the variation of top displacement, top acceleration and objective function (linear combination of top displacement and top acceleration). The phase of the ground motion is that of El Centro NS 1940. Since the parameters in Eq. (1) are specified as *a* = 1, *b* = 1, the objective function for the initial model is 2.0. No clear tendency of the reduction of story stiffness is seen (like the soft first-story type observed for the hybrid system) and the top displacement increases gradually from the beginning. This result indicates that the proposed algorithm does not work well for the building without connecting mechanism and the proposed algorithm is suitable for the proposed smart hybrid system with the connecting damper system and the base-isolation system.

### 4.4 Energy response to simulated high-frequency ground motion

**Fig. 11:**
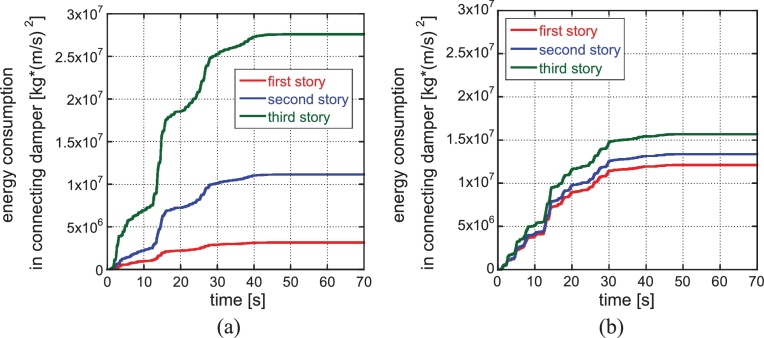
Energy consumption at connecting dampers in the 1st, 2nd and 3rd stories for the initial model and the soft first-story model: (a) Initial model, (b) Soft first-story model (phase: El Centro NS 1940) (‘story’ is used here to designate the element of stiffness in reduced three-mass model).

**Fig. 12:**
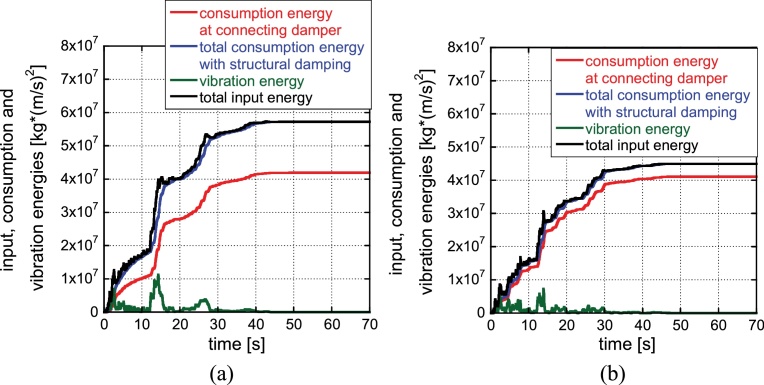
Energy time histories for the initial model and the soft first-story model: (a) Initial model, (b) Soft first-story model (phase: El Centro NS 1940).

Fig. 11 shows the energy consumptions at connecting dampers in the 1st, 2nd and 3rd stories for the initial model (Fig. 11(a)) and the automatically generated soft first-story model (Fig. 11(b)) under the ground motion of the phase of El Centro NS 1940. It can be understood that almost uniform energy consumption at connecting dampers is realized in all stories in the soft first-story model. Furthermore, Fig. 12 presents the energy time histories for the initial model (Fig. 12(a)) and the soft first-story model (Fig. 12(b)) under the ground motion of the phase of El Centro NS 1940. It can be found that the total input energy to the whole hybrid system, the total consumption energies at the connecting dampers, the total consumption energies at the structures and the vibration energy (kinetic energies and elastic strain energies in structures) in the soft first-story model are smaller than those in the initial model although the consumption energy at the connecting dampers is almost constant regardless of the models.

The response of the adjacent structure (free wall) is not important because the design margin for safety in the adjacent structure is rather large compared to the main structure and such response does not exhibit undesirable response compared to the connected model without base-isolation system and the unconnected model. Although the model parameters are somewhat different from the present model, the responses of adjacent structures are shown in the previous paper (Figs. 19 and 20 in Murase et al. (2013)).

### 4.5 Overall assessment of proposed hybrid system against single-mechanism models via transfer function

In the previous sections, the performance assessment of the proposed hybrid system has been conducted principally for the ground motions with rather high frequencies. In order to demonstrate more general properties of the proposed hybrid system for a broader range of frequency, the transfer functions are shown in this section.

**Fig. 13:**
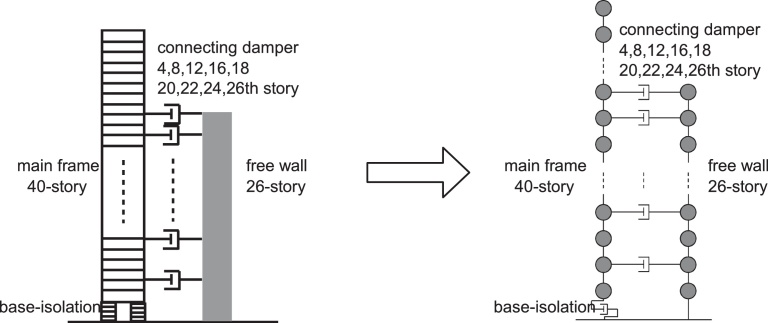
Hybrid system consisting of a 40-story base-isolated main building, a free wall of 26 stories and a set of interconnecting oil dampers (Murase et al., 2013).

Consider a 40-story base-isolated main building, a free wall of 26 stories and a set of oil dampers as shown in Fig. 13. The oil dampers are installed at 4, 8, 12, 16, 18, 20, 22, 24 and 26th floor levels. The floor mass of the main building is 1.7 × 10^6^ (kg) and that of the free wall is 2.2 × 10^5^ (kg). The base-isolation floor mass is 5.1 × 10^6^ (kg). The story height is 3.5(m) in all the stories. The super-structure of the main building is designed so as to have the fundamental natural period of 3.0(s) and a straight fundamental mode for a fixed base model. On the other hand, the free wall is designed so as to have the fundamental natural period of 0.63(s) and a straight fundamental mode.

The fundamental natural period of the hybrid system is 6.72(s). The higher-mode natural periods of the hybrid system are 1.72(s), 0.965(s), 0.672(s) and 0.640(s). The fundamental natural period (0.63s) of the free wall corresponds to the natural period 0.640(s) of the 5th mode of the hybrid system. The structural damping ratio of the super-structure (stiffness-proportional damping) is set to 0.03 and the damping ratio of the base-isolation story for a rigid super-structure is 0.15. The oil dampers are allocated uniformly to the specific floors mentioned above and the approximate lower-mode damping ratio for a rigid free wall is set to 0.15 under non-modal-coupling approximation.

**Fig. 14:**
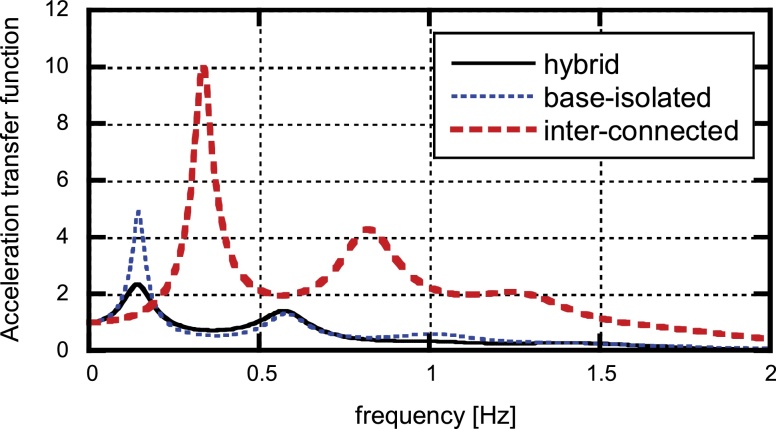
Acceleration transfer functions at top of main frame for the hybrid system, the base-isolated model without interconnection and the interconnecting model without base-isolation (Murase et al., 2013).

**Fig. 15:**
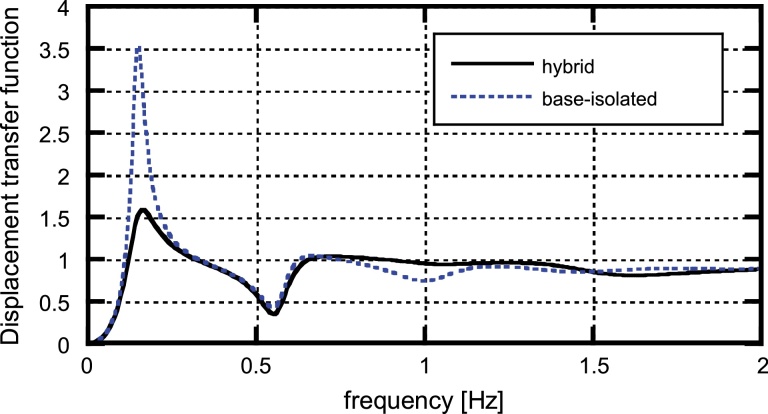
Displacement transfer functions (deformation of base-isolation story) for the hybrid system and the base-isolated model without interconnection (Murase et al., 2013).

The transfer characteristics of the present hybrid system to the base input are shown here. Fig. 14 shows the acceleration transfer functions at the top of the main frame for the hybrid system, the base-isolated model without interconnection and the interconnecting model without base-isolation. On the other hand, Fig. 15 presents the displacement transfer functions (deformation of base-isolation story) for the two models including the base-isolation story among three. It can be observed that the hybrid system is superior to other two single-mechanism models (base-isolated building and connected buildings without base-isolation) both in the acceleration and displacement transfer properties. Especially the hybrid system possesses an effective control performance at the fundamental natural period of the base-isolated main building. It can also be found that the lowest two eigenmodes are predominant. This fact supports the validity of the simplification of the original model into the model with three degrees of freedom in Section 4.2.

## 5 Conclusions

The following conclusions have been derived.

(1) An automatic generation algorithm of the proposed smart base-isolation and building-connection hybrid system has been proposed.
(2) It has been demonstrated that, once an objective function in terms of top displacement and top acceleration under a design ground motion is introduced and a sensitivity-based algorithm is devised, a smart hybrid system consisting of a base-isolation system and a building connection system can be generated automatically.
(3) While the proposed algorithm does not work well in a building without the connecting-damper system, it works well in the proposed smart hybrid system with the connecting damper system. The smart hybrid system has a soft first-story mechanism and the mechanism indicates that the automatic introduction of the base-isolation system is possible and desired in the main structure from the viewpoint of performance upgrade.
(4) It has been made clear from the energy analysis that the proposed smart hybrid system makes the connecting damper at every floor level effective.

## Declarations

### Author contribution statement

Masatoshi Kasagi: Conceived and designed the experiments; Analyzed and interpreted the data; Wrote the paper.

Kohei Fujita, Masaaki Tsuji: Analyzed and interpreted the data.

Izuru Takewaki: Conceived and designed the experiments; Wrote the paper.

### Funding statement

This work was supported by Grant-in-Aid for Scientific Research of Japan Society for the Promotion of Science (No.15H04079).

### Competing interest statement

The authors declare no conflict of interest.

### Additional information

No additional information is available for this paper.

## Figures and Tables

**Table 1:** Parameters of main building and free wall.

	Main structure	Free wall
Number of stories	30	30
Floor mass (kg)	900 × 10^3^	144 × 10^3^
Fundamental natural period (s)	2.4	1.0
Structural damping ratio (stiffness-proportional)	0.03	0.03

**Table 2:** Simplified three-mass model (‘story’ is used here to designate the element of stiffness in reduced three-mass model).

		Main structure	Free wall
Mass at each node (kg)		9000 × 10^3^	1440 × 10^3^
Story stiffness (N/m)	Third story	1.85 × 10^8^	1.71 × 10^8^
Second story	3.08 × 10^8^	2.84 × 10^8^
First story	3.70 × 10^8^	3.41 × 10^8^
Fundamental natural period (s)		2.4	1.0

## References

[bib0005] Architectural Institute of Japan (AIJ) (2011). Structural design with higher robustness and redundancy. J. Archit. Build. Sci..

[bib0010] Ariga T., Kanno Y., Takewaki I. (2006). Resonant behavior of base-isolated high-rise buildings under long-period ground motions. Struct. Des. Tall Spec. Build..

[bib0015] Ben-Haim Y. (2001). Infomation-gap decision theory: Decisions under severe uncertainty.

[bib0020] Elishakoff I., Ohsaki M. (2010). Optimization and anti-optimization of structures under uncertainty.

[bib0025] Hall J.H., Heaton T.H., Halling M.W., Wald D.J. (1995). ‘Near-source ground motion and its effect on flexible buildings’. Earthq. Spectra.

[bib0030] Heaton T.H., Hall J.H., Wald D.J., Halling M.W. (1995). Response of high-rise and base-isolated buildings in a hypothetical MW 7 · 0 blind thrust earthquake. Science.

[bib0035] Hino J., Yoshitomi S., Tsuji M., Takewaki I. (2008). Bound of aspect ratio of base-isolated buildings considering nonlinear tensile behavior of rubber bearing. Struct. Eng. Mech..

[bib0040] Irikura K., Kamae K., Kawabe H. (2004). Importance of prediction of long-period ground motion during large earthquakes. Annual Conference of the Seismological Society of Japan, Poster session.

[bib0045] Jangid R.S. (1995). Optimum isolator damping for minimum acceleration response of base-isolated structures. Aust. Civil Eng. Transactions.

[bib0050] Jangid R.S., Banerji P. (1998). Effects of isolation damping on stochastic response of structures with nonlinear base isolators. Earthq. Spectra..

[bib0055] Jangid R.S., Datta T.K. (1994). Non-linear response of torsionally coupled base isolated structure. J. Struct. Eng. ASCE.

[bib0060] Jangid R.S., Kelly J.M. (2001). Base isolation for near-fault motions. Earthq. Eng. Struct. Dyn..

[bib0065] Kamae K., Kawabe H., Irikura K. (2004). Strong ground motion prediction for huge subduction earthquakes using a characterized source model and several simulation techniques. Proceedings of the 13th WCEE.

[bib0070] Karabork T. (2011). Performance of multi-storey structures with high damping rubber bearing base isolation systems. Struct. Eng. Mech..

[bib0075] Kelly J.M. (1999). The role of damping in seismic isolation. Earthq. Eng. Struct. Dyn..

[bib0080] Koo J.-H., Jang D.-D., Usman M., Jung H.-J. (2009). A feasibility study on smart base isolation systems using magneto-rheological elastomers. Struct. Eng. Mech..

[bib0085] Li H.-N., Wu X. (2006). Limitations of height-to-width ratio for base-isolated buildings under earthquake. Struct. Des. Tall Spec. Build..

[bib0090] Morales C.A. (2003). Transmissibility concept to control base motion in isolated structures. Eng. Struct..

[bib0095] Murase M., Tsuji M., Takewaki I. (2013). Smart passive control of buildings with higher redundancy and robustness using base-isolation and inter-connection. Earthq. Struct..

[bib0100] Naeim F., Kelly J.M. (1999). Design of Seismic Isolated Structures.

[bib0105] Nishimura K., Fukumoto Y., Wada Y. (2008). Response control effect of hi-rised reinforced concrete building using couplaed vibration control structure. J. Technol. Des. AIJ.

[bib0110] Patel C.C., Jangid R.S. (2011). Dynamic response of adjacent structures connected by friction dampers. Earthq. Struct..

[bib0115] Petti L., De Iuliis G.G.M., Palazzo B. (2010). Small scale experimental testing to verify the effectiveness of the base isolation and tuned mass dampers combined control strategy. Smart Struct. Syst..

[bib0120] Takewaki I. (2005). Uncertain-parameter sensitivity of earthquake input energy to base-isolated structure. Struct. Eng. Mech..

[bib0125] Takewaki I. (2006). Critical excitation methods in earthquake engineering.

[bib0130] Takewaki I. (2007). Earthquake input energy to two buildings connected by viscous dampers. J. Struct. Eng. ASCE.

[bib0135] Takewaki I. (2008). Robustness of base-isolated high-rise buildings under code-specified ground motions. Struct. Des. Tall Spec. Build..

[bib0140] Takewaki I. (2013). Toward greater building earthquake resilience using concept of critical excitation: a review. Sust. Cities Soc..

[bib0145] Takewaki I., Fujita K. (2009). Earthquake input energy to tall and base-isolated buildings in time and frequency dual domains. Struct. Des. Tall Spec. Build..

[bib0150] Takewaki I., Tsujimoto H. (2011). Scaling of design earthquake ground motions for tall buildings based on drift and input energy demands. Earthq. Struct..

[bib0155] Takewaki I., Murakami S., Fujita K., Yoshitomi S., Tsuji M. (2011). The 2011 off the Pacific coast of Tohoku earthquake and response of high-rise buildings under long-period ground motions. Soil Dyn. Earthq. Eng..

[bib0160] Takewaki I., Moustafa A., Fujita K. (2012). Improving the earthquake resilience of buildings: The worst case approach.

[bib0165] Takewaki I., Fujita K., Yoshitomi S. (2011). Uncertainties in long-period ground motion and its impact on building structural design: Case study of the Tohoku (Japan) earthquake. Eng. Struct..

[bib0170] Takewaki I., Lagaros N.D., Tsompanakis Y., Papadrakakis M. (2015). Fundamental properties of earthquake input energy on single and connected building structures. New Trends in Seismic Design of Structures edited by.

[bib0175] Trombetti T., Silvestri S. (2004). Added viscous dampers in shear-type structures: The effectiveness of mass proportional damping. J. Earthq. Eng..

[bib0180] Xu Z., Agrawal A.K., He W.L., Tan P. (2007). Performance of passive energy dissipation systems during near-field ground motion type pulses. Eng. Struct..

